# The anti-inflammatory and tolerogenic potential of small spleen peptides

**DOI:** 10.3389/fimmu.2024.1449657

**Published:** 2024-09-02

**Authors:** Viktor Wixler, Igor Z. Zaytsev, Yvonne Boergeling, Stephan Ludwig

**Affiliations:** ^1^ Institute of Molecular Virology, Centre for Molecular Biology of Inflammation, Westfaelische Wilhelms-University, Muenster, Germany; ^2^ Institute of Pharmaceutical Technologies, Moscow, Russia

**Keywords:** peripheral immune tolerance, dendritic cells, Treg cells, mTOR signaling cascade, extracellular ATP, adenosine, tolerogenesis, immunogenesis

## Abstract

Maintaining peripheral immune tolerance and preventing harmful autoimmune reactions is a fundamental task of the immune system. However, these essential functions are significantly compromised during autoimmune disorders, creating a major challenge in treating these conditions. In this context, we provide an overview of research on small spleen polypeptides (SSPs) that naturally regulate peripheral immune tolerance. Alongside outlining the observed effects of SSPs, we summarize here the findings on the cellular and molecular mechanisms that underlie their regulatory impact. Specifically, SSPs have demonstrated remarkable effectiveness in halting the progression of developing or established autoimmune disorders like psoriasis or arthritis in animal models. They primarily target dendritic cells (DCs), swiftly prompting the production of extracellular ATP, which is then degraded and sensed by adenosine receptors. This process triggers the mTOR signaling cascade, similar to powerful immune triggers, but instead of a rapid and intense reaction, it leads to a moderate yet significant activation of the mTOR signaling cascade. This induces a tolerogenic state in dendritic cells, ultimately leading to the generation of Foxp3^+^ immunosuppressor Treg cells. In addition, SSPs may indirectly attenuate the autoimmune response by reducing extracellular ATP synthesis in non-immune cells, such as endothelial cells, when exposed to elevated levels of proinflammatory cytokines. SSPs thus have the potential to contribute to the restoration of peripheral immune tolerance and may offer valuable therapeutic benefits in treating autoimmune diseases.

## Introduction

The causes and forms of autoimmune diseases are numerous and diverse. Very often, it begins with physical or mental stress (severe insult, violence, loss of loved ones), poisoning (e.g., with heavy metals or aggressive organic substances), parasitic invasions, viral, bacterial, or fungal infections. Such factors place a significant burden on the immune system and may lead to sustained activation of autoimmunity. Under normal circumstances, the immune system can manage moderate stress through internal regulatory mechanisms that restore immune balance, known as homeostasis. However, if the body is regularly or continuously exposed to provocateurs that maintain inflammatory stress, the pressure on the immune system becomes too massive, leading to impaired immune tolerance and autoimmune disorders.

The logical conclusion from this is to stimulate the immune system with drugs that restore the disturbed homeostasis. However, presently, only drugs that target symptoms rather than the cause, such as non-steroidal anti-inflammatory drugs, immunosuppressants (cyclosporine, methotrexate, azathioprine) and glucocorticosteroids (GCS) have been employed for this purpose (1, 2). Unfortunately, their long-term use often leads to more severe side effects than the burden caused by the underlying disease, including opportunistic infections, myopathy, gastrointestinal issues, and organ failure (3, 4). More recently, monoclonal antibodies targeting inflammatory cytokines and their receptors have been utilized, which affect individual inflammatory factors but do not eliminate the cause of inflammation and, thus, also only have symptomatic effects (5–7).

Given the immune system’s innate ability to restore balance, it is evident that in severe autoimmune diseases, this capability should be reactivated through an external pharmacological stimulus. This stimulus should mimic the natural process and should have the ability to induce peripheral tolerogenesis, thereby aiding in the rebalancing of the immune system. Such an approach would represent an ideal solution, particularly for severe autoinflammation or autoimmune diseases.

The spleen is both a secondary lymphoid organ and the largest immune organ in the body, playing a vital role in immune function and overall health (8, 9). It acts as an important filter for pathogens and antigens in the blood, contributes significantly to iron metabolism and erythrocyte homeostasis, and serves as a reservoir for immune cells (10–12). In addition, the spleen plays a crucial role in both innate and adaptive immune responses, maintaining peripheral immune homeostasis and complementing central immune tolerance (9, 10, 12). In line with this, some publications have highlighted the positive immunostimulatory and detoxifying effects of extracorporeal perfusion of donor or porcine xenospleen in patients with specific diseases such as systemic lupus erythematosus or septic complications (13–15). Spleen transplantation has also been reported to have a tolerogenic effect in some rodent models (16).

Although the spleen undoubtedly plays a central role in the development of autoimmune diseases, the effects of spleen removal on the progression of these diseases are often complex. Studies in animal models have shown that mice or rats that underwent splenectomy developed autoimmune diseases similar to the control group, but with changes in the cellular and humoral immune responses that were more multidirectional, probably due to the compensatory effect of the lymph nodes (17–19). Clinical studies have also linked splenectomy to the development of new autoimmune phenomena and to changes in the clinical course of patients with pre-existing autoimmune diseases. For example, one case report documented the occurrence of glomerulonephritis and antiphospholipid syndrome following elective splenectomy in a patient previously diagnosed with autoimmune hepatitis and rheumatoid arthritis (20). The complex effects of splenectomy may be influenced by the specific autoimmune disease, individual patient characteristics and potential compensatory mechanisms involving other peripheral lymphoid organs.

Recently, some published studies have shown that the spleen naturally contains small polypeptides with immunomodulatory properties (21–23). These polypeptides are able to stimulate the proliferation of several immune cells, induce the production of important cytokines such as IL-2, IL-4, IFNγ and TNFα, and increase the levels of immunoglobulins (21). When administered to mice, these polypeptides were able to increase their survival rate after influenza A virus infection by enhancing T lymphocyte-dependent defense mechanisms against influenza A virus (23). In addition, these small polypeptides have been shown to counteract autoimmune activation and have the potential to regulate autoimmune diseases (22, 24). The latent potential of the spleen in this regard is eagerly awaiting activation. This review aims to consolidate our understanding of the effect of SSPs in suppressing the onset of autoimmune diseases *in vivo*, while exploring the underlying cellular and molecular mechanisms.

## Phenomenology: *in vivo* anti-inflammatory effects of SSPs

In search for natural regulators of peripheral immune tolerance, the entire pool of spleen proteins was meticulously fractionated according to their molecular weight. Surprisingly, only the fraction with a molecular weight of less than 12 kDa showed a significant anti-inflammatory effect. Conversely, the higher molecular weight protein fraction, which is expected to contain most of the regulatory immune cytokines found in the spleen, failed to demonstrate any effect (22).

Administering the small splenic polypeptide fraction to transgenic ihTNFtg mice, who were experiencing severe psoriatic arthritis triggered by elevated hTNFα cytokine levels following doxycycline stimulation (25), led to a significant inhibition of both psoriasis and arthritis (22). Notably, the SSPs not only did impede the onset and progression of psoriatic arthritis but also halted the progression of an already existing hyperinflammatory disease. Particularly remarkable was the fact that these outcomes occurred despite the persistent high levels of soluble hTNFα in the bloodstream and affected tissues, emphasizing the potent anti-inflammatory properties of SSPs. It is noteworthy that the source of the polypeptides, whether from the spleen of pigs, cattle, or mice, was inconsequential for their function (22, 24).

The reduction in autoimmune disease severity mediated by SSPs was associated with a decreased expression of inflammatory markers such as S100A9 and Ki-67, as well as a reduction in immune cell infiltration in affected skin and joint tissues (22). Additionally, there was an increase in the number of CD25^+^/CTLA4^+^/Foxp3^+^ inhibitory CD4 Treg cells in the regional lymph nodes. It’s worth noting that depleting the CD25-positive pool of CD4 Treg cells in the mice reversed the anti-inflammatory action of SSPs. Remarkably, all of these effects occurred despite the presence of extremely high levels of the cytokine hTNFα (22). These findings emphasize the complex impact of SSP in alleviating autoimmune diseases, encompassing the modulation of inflammatory markers, immune cell infiltration, and the balance of regulatory T cells.

## SSPs target dendritic cells

The anti-inflammatory effect of SSPs, coupled with the increase in Treg cells, suggests that immune cells are the primary targets of SSPs. However, when macrophages or T lymphocytes were directly stimulated with SSPs *in vitro*, no effect on their activation was observed. Furthermore, in ihTNFtg/Rag1^KO^ mice, which lack mature T and B lymphocytes but have an intact innate immune system, SSPs did not exhibit an anti-inflammatory effect (22). These mice develop a more severe disease than ihTNFtg mice due to an increased infiltration of inflamed sites with macrophages (26). However, treatment with SSPs did not alter skin or paw inflammation or hTNFα expression in ihTNFtg/Rag1^KO^ mice. This suggests that SSPs primarily affect the adaptive rather than the innate immune system and that T cells are not directly affected.

As the activation of T cells, including the immunosuppressive Foxp3^+^ Treg cells, hinges on the involvement of dendritic cells (DCs) and their antigen-presenting MHCII receptor, along with co-stimulatory molecules like CD80/86 (Cluster of Differentiation 80/86), CD40, PD-L1/2, or CD205, these cells were subjected to detailed investigation. Remarkably, SSPs were found to induce the expression of tolerogenic receptors such as PD-L1 and CD205 in immature DCs, steering them toward a tolerogenic differentiation (22). Notably, this effect was more pronounced in cases where both DCs and CD4 T cells were of syngeneic sources with minimal antigenic disparities, as opposed to allogeneic sources with higher antigenicity. This suggests that SSPs exhibit efficiency in fine-tuning immune responses, particularly in scenarios involving low-threshold antigenic differences commonly seen in chronic autoimmune diseases. In addition, SSPs were as efficient as classical tolerogenic inducers such IL-10 or TGFβ in their ability to trigger tolerogenesis of immature DCs, but the mechanism of DC activation by SSPs was different from that of IL-10 or TGFβ and did not mimic the apoptosis-mediated tolerogenic stimulation (22, 24, 27). SSPs primarily target DCs and induce a tolerogenic state that leads to the induction of Foxp3^+^ immunosuppressor Treg cells and the latter requires direct contact between SSP-activated DCs and naive CD4^+^ T cells via PD-1 and CTLA4 immune checkpoint receptors of T cells (22). Thus, SSPs are natural splenic polypeptides with the ability to restore impaired peripheral tolerance and prevent autoimmune diseases without the obvious side effects usually associated with the use of IL-10 or TGFβ (28, 29), which makes SSPs extremely attractive as an anti-inflammatory medication.

## Thymosins are the main components of SSPs

Obviously, a critical issue related to the described phenomena was the identification of active substances in the SSP samples. Consequently, a tandem mass spectrometry analysis was conducted on the SSP samples, unveiling thymosins as the primary constituents of the SSP preparations. Thymosin beta 4, thymosin beta 10, parathymosin, and prothymosin alpha were identified, with thymosin beta 4 (Tβ4) emerging as the most abundant polypeptide, surpassing other thymosin forms by 1 to 2 orders of magnitude in content (24). This discovery was both unexpected and exciting, considering that these small polypeptides are widely distributed in all animal tissues, and are recognized for their diverse biological effects. Tβ4 is highly conserved across a wide range of organisms, from invertebrates to mammals, and was originally identified as a protein that binds to G-actin, preventing its polymerization (30). Tβ4 is not only localized inside cells, but can also be released into the extracellular space by still unknown mechanisms. In mammals, highest levels were demonstrated in the spleen (31). Apart from regulating cytoskeletal dynamics, Tβ4 is implicated in cell migration, tissue remodeling, angiogenesis, and the recruitment of stem cells to injured tissues (30, 32). Furthermore, it demonstrates anti-inflammatory properties and shields cells from oxidative stress. Studies have also suggested a possible role of Tβ4 in the pathogenesis of rheumatoid arthritis (RA), as significant increases in Tβ4 levels were observed in the synovial fluid and serum of RA patients, which appear to prevent the activation of immune responses associated with RA (33). Despite its short length of only 43 amino acids (5 kDa), the polypeptide undergoes various post-translational modifications, including glycosylation, acetylation or phosphorylation at numerous sites. Additionally, an N-terminal peptide of four amino acids known as Ac-SDKP can be liberated from Tβ4 through enzymatic hydrolysis, leading to the removal of the first methionine and the N-acetylation of the peptide. Consequently, secondary modifications of Tβ4 and its Ac-SDKP derivative have been recognized to be crucial for the biological activities of the Tβ4 molecule (32, 34).

Surprisingly, despite the well-documented role of Tβ4 in diverse physiological processes like wound healing, tissue regeneration, and immunomodulation, its purified recombinant samples demonstrated decreased effectiveness as anti-inflammatory medications *in vivo* when compared to SSP samples (24). It is plausible that naturally derived SSPs contain unique thymosin variants that synergistically enhance each other’s effects, or that post-translational modifications crucial for biological activity are insufficiently present in recombinant or chemically synthesized samples. It is also conceivable that a combination of both factors contributes to this disparity. Consequently, these results not only confirm but also emphasize the vital role of SSPs as natural regulators of peripheral immunological tolerance *in vivo*.

## Molecular mechanism of SSP action

The protein structure of thymosins closely resembles that of the inhibitory factor 1 (IF1) of ecto-ATP synthase, which is a binding factor of this membrane enzyme (35). IF1 functions to inhibit excessive ATP hydrolysis by binding to the F1 component of ecto-ATP synthase, thereby impeding its backward movement and the hydrolysis of ATP. More recent findings, however, indicate that IF1 can also influence ATP synthesis activity (36, 37). Additionally, it has been shown that Tβ4 binds strongly to the α and β subunits of membrane ATP synthase and, like IF1, affects the function of ecto-ATP synthase (35). It is worth noting that both thymosins and extracellular ATP (exATP) are recognized for their influence on immune response activity (32, 38).

And indeed, SSPs had proven to be crucial regulators of cellular exATP levels (24). Real-time monitoring of ATP content in immature DCs revealed that SSPs induce a significant *de novo* synthesis of exATP. This synthesis peaked approximately two hours after the onset of stimulation, followed by rapid degradation. Interestingly, the behavior of SSPs resembled that of other tolerogenic stimuli such as IL-10 or TGFβ, but differed significantly from that of immunogenic stimuli such as LPS or GM-CSF+IL-4. The latter factors only induced a moderate *de novo* synthesis of exATP, albeit with delayed degradation.

The significant differences in exATP synthesis and degradation have noteworthy biological implications. Of particular importance in this context is the ATP degradation product adenosine, acknowledged as a crucial tolerogenic stimulus, unlike ATP which is typically seen as a “danger signal” that fosters immune responses (38–41). The experiments indicate that the difference in adenosine levels on the DC surface resulting from tolerogenic SSPs or immunogenic factors is relatively small, only 2 to 3-fold (24). However, this subtle variation ultimately played a decisive role in determining the fate of the DCs, whether they further specialized towards tolerogenesis or immunogenesis.

This conclusion was further reinforced by the inhibition of adenosine receptors on DCs during their stimulation with SSPs or other tolerogenic stimuli. Blocking adenosine receptors resulted in a notable decrease in their capacity to promote tolerogenic Foxp3^+^ Treg cells. However, this blockade did not impact the proliferation of CD4^+^ cells or their differentiation into immunogenic Tbet^+^ Th1 cells (24).

Similar to other cells, the differentiation of immature DCs into specialized immunogenic or tolerogenic cells is governed by specific stimuli that activate intracellular signaling pathways. In this context, the mTOR (mammalian target of rapamycin) signaling pathway appeared to play a central role in the SSP-mediated tolerogenic development of DCs (27). In-depth analysis of mTOR pathway activation by SSPs showed that, contrary to the prevailing notion in the literature that this pathway is only activated in DCs during immunogenesis and repressed during tolerogenesis, the mTOR cascade is robustly activated during tolerogenic stimulation, only the mode of activation differs from that of immunogenic stimulation (27). The disparity in mTOR activation between immunogenic and tolerogenic stimuli is quantitative rather than qualitative. While immunogenic activation is rapid, strong, and sustained, the activity induced by tolerogenic SSPs is delayed, less intense, yet still significant. In both cases, mTOR activation primarily occurs through the PI3K/Akt signaling axis and involves ERK and GSK3β kinases, with a minimal involvement of AMPK or NF-kB pathways (27). While the activation kinetics of the mTOR cascade after stimulation with SSPs did not differ from that of the other tolerogenic stimuli, the initiation of this activation appears to be different. In the case of SSPs, mTOR activation seems to involve adenosine receptors, unlike in the case of LPS-stimulated immunogenesis. Both the induction of the mTOR cascade in DCs (27) and the resulting expression of tolerogenic markers (22) differed from those induced by IL-10 or TGFβ, underscoring the unique nature of SSPs.

Interestingly, among adenosine receptors, only A1 and A3, rather than A2A and A2B, appear to play a critical role in SSP-mediated tolerogenesis of DCs, as only their engagement led to the activation of the mTOR signaling cascade (27). This finding aligns with results indicating that the inhibition of PKA does not significantly affect SSP-induced mTOR activation. It should be noted in this context that, while all four adenosine receptors belong to the class of purinergic G-protein-coupled receptors, they signal differently. The A2A and A2B subtypes primarily signal via Gs proteins, resulting in the activation of adenylyl cyclase and the stimulation of cyclic adenosine monophosphate (cAMP) formation, as well as the activation of PKA. Conversely, the A1 and A3 subtypes signal via Gi proteins, leading to the inhibition of adenylyl cyclase and PKA (42, 43).

## SSPs reduce inflammatory cytokine-induced exATP synthesis in non-immune cells

The primary aim of this review was to consolidate the existing findings on SSPs as natural regulators of peripheral immunological tolerance, spanning the phenomenology at the organism level during autoimmune diseases and the underlying cellular and molecular mechanisms. However, given that SSP samples consist primarily of thymosins, particularly Tβ4, which is known for its targeting of various cells and the multitude of biological effects it exerts, it was reasonable to speculate that SSPs might have additional effects. These additional effects may indirectly influence the regulation of immune responses mediated by thymosins, a topic we will briefly address here. This includes the role of Tβ4 in corneal wound healing, where it promotes epithelial cell migration, reduces inflammation, and inhibits apoptosis (44). Furthermore, it contributes to the survival and angiogenesis of transplanted endothelial progenitor cells in the infarcted myocardium and takes part in the regulation of hair growth (32, 45–47). Particularly noteworthy is its capacity to influence the development of microvessel endothelial cells and mural cells, thereby contributing to vascular wall stability (48–50). This is significant because microvessel endothelial cells play a key role in microcirculatory diseases such as thrombotic microangiopathies and diffuse intravascular coagulation, and their activation is an important feature in these conditions (51). Additionally, endothelial cells are crucially involved in maintaining blood fluidity and providing controlled vascular hemostasis at sites of injury, thereby facilitating multiple mechanisms that must be kept in balance (52).

**Figure 1:**
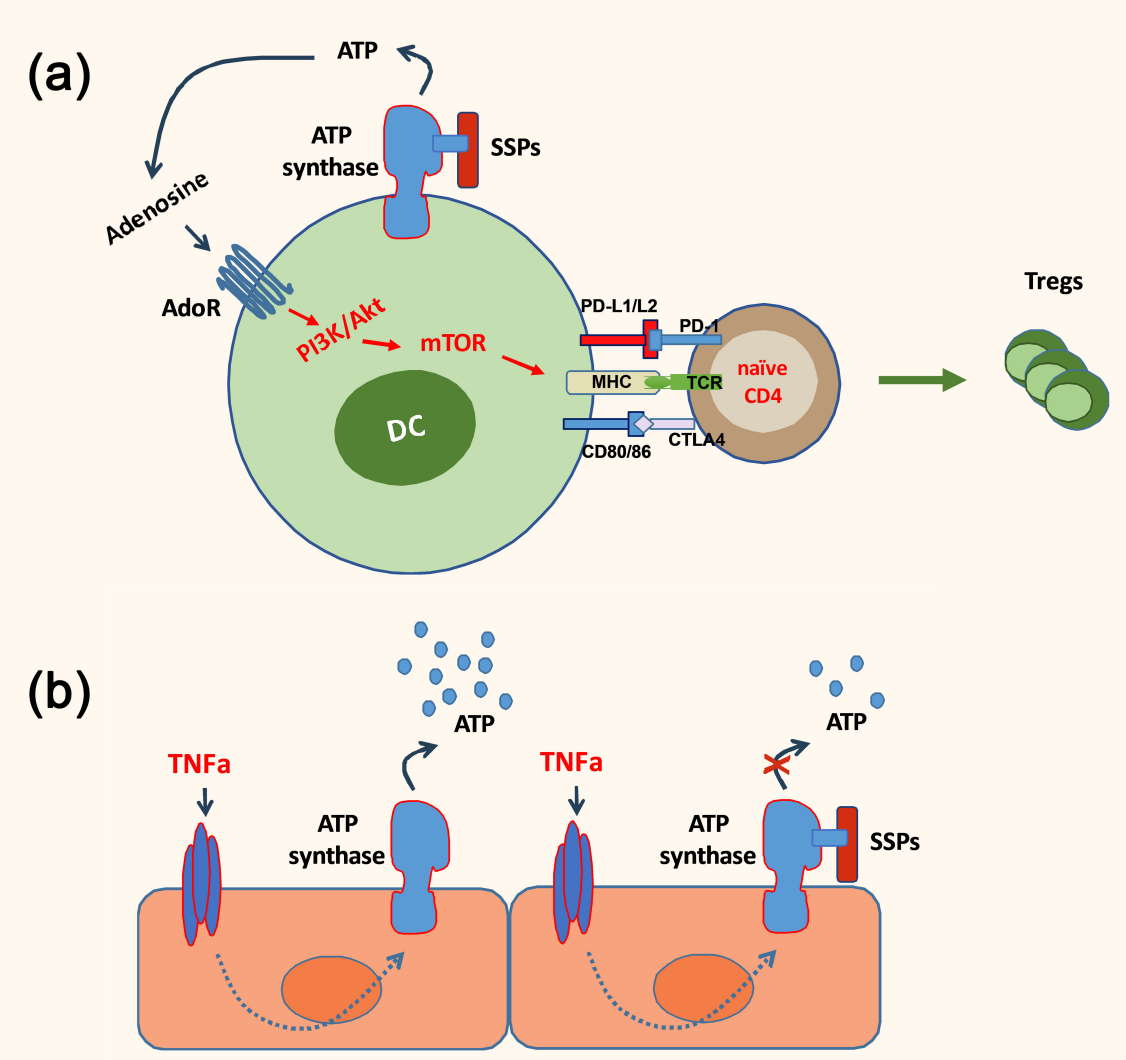
A simplified scheme depicting the anti-inflammatory action of SSPs. SSPs may exert a direct influence on the immune response by inducing the conversion of dendritic cells into tolerogenic cells, thereby facilitating the generation of Foxp3^+^ Treg cells **(A)**. Additionally, they can indirectly modulate the immune response by reducing the exATP synthesis in non-immune cells, such as endothelial cells, which is induced by proinflammatory cytokines **(B)**.

Thus, considering that Tβ4 is the most prevalent component of SSP samples and regulates the synthesis and degradation of exATP in DCs, it was explored whether these anti-inflammatory, tolerogenic agents could also diminish ATP release in non-immune cells. Specifically, the focus was on cells lining smaller blood vessels, which are the primary targets for soluble pro-inflammatory cytokines and foreign immune substances such as LPS, thereby contributing to chronic tissue inflammation. These studies have unveiled that the regulation of exATP levels during inflammatory conditions appears to be a shared characteristic of SSPs. SSPs demonstrated a decrease in exATP synthesis in various cell types, including synovial fibroblasts from arthritis patients, keratinocytes and mural cells, when exposed to proinflammatory factors (24). This effect was particularly pronounced in primary endothelial cells and pericytes, highlighting the anti-inflammatory properties of SSPs. Hence, it seems that SSPs play a role in diminishing autoimmune responses through direct modulation of DCs via the stimulation of their exATP synthesis and degradation, as well as indirectly by attenuating ATP synthesis in non-immune cells triggered by proinflammatory cytokines (**Figure 1**).

## Conclusions and future perspectives

Collectively, the studies summarized here unequivocally demonstrate the tolerogenic properties of SSPs (22, 24, 27). They showed high efficacy *in vivo* and proved to be as effective in generating immunosuppressive Treg cells as canonical tolerogenic factors such as IL-10 and TGFβ. SSPs seem to achieve their anti-inflammatory and tolerogenic function by regulating the synthesis of exATP and its conversion to adenosine. While the timing of exATP induction and degradation, as well as the activation kinetics of the mTOR signaling cascade mirrored those of canonical IL-10 and TGFβ stimuli, the mechanisms of DC stimulation were distinct. They were able to exert their anti-inflammatory effects even in the presence of high concentrations of proinflammatory cytokines. Furthermore, their use as natural regulators of tolerance development is expected to lead to reduced inflammatory side effects as they do not trigger phosphorylation of STAT or SMAD proteins, which are typical for IL-10 and TGFβ stimuli and are associated with inflammation. This, combined with the fact that SSPs actively reduce the harmful exATP levels induced by pro-inflammatory stimuli in non-immune cells such as endothelial cells and pericytes and their potential ability to regulate vascular tone, makes them attractive candidates for drug development, especially considering that SSPs showed better anti-arthritis activity than pure Tβ4. Finally, considering the remarkable success of SSPs in combating autoimmune disorders in laboratory animals, it seems promising to investigate the combined efficacy of SSPs with anti-inflammatory treatments aimed at reducing various autoimmune triggers and thus enhancing the anti-inflammatory effect of SSPs, and to initiate clinical trials as soon as possible.
